# Role of bridging therapy during chimeric antigen receptor T cell therapy

**DOI:** 10.1002/jha2.335

**Published:** 2021-11-19

**Authors:** Shakthi T. Bhaskar, Bhagirathbhai R. Dholaria, Salyka M. Sengsayadeth, Bipin N. Savani, Olalekan O. Oluwole

**Affiliations:** ^1^ Division of Hematology/Oncology Department of Medicine Vanderbilt University Medical Center Nashville Tennessee USA

**Keywords:** bridging therapy, chimeric antigen receptor T cell therapy, hematologic malignancies

## Abstract

Chimeric antigen receptor (CAR) T‐cell therapy has been approved for use in several relapsed/refractory hematologic malignancies and has significantly improved outcomes for these diseases. A number of different CAR T products are now being used in clinical practice and have demonstrated excellent outcomes to those in clinical trials. However, increased real‐world use of CAR T therapy has uncovered a number of barriers that can lead to significant delays in treatment. As a result, bridging therapy has become a widely used tool to stabilize or debulk disease between leukapheresis and CAR T cell administration. Here we review the available data regarding bridging therapy, with a focus on patient selection, choice of therapy, timing of therapy, and potential pitfalls.

## 1 INTRODUCTION

Chimeric antigen receptor (CAR) T‐cell therapy has been an emerging area of interest in cancer therapeutics since first being proposed in the 1980s. Initial small clinical studies were conducted in the early 2000s but results at the time were not promising, due in large part to a limited persistence and in vivo expansion of these cells [1]. More recently, however, the integration of costimulatory domains in the engineering of CAR T‐cells has led to significant increases in the efficacy of this therapy [2]. As a result of these advances, several clinical trials emerged to evaluate the safety and efficacy of CAR T therapy in a variety of malignancies.

Adults with relapsed/refractory acute lymphoblastic leukemia (ALL), diffuse large B‐cell lymphoma (DLBCL), and mantle cell lymphoma (MCL) have a poor prognosis. Relapsed/refractory ALL is associated with a median overall survival (OS) of approximately 8 months. Newer therapies such as the CD19‐targeted bispecific T cell engager (blinatumomab) and CD22 antibody drug conjugate (Inotuzumab ozogamicin) have expanded salvage therapy options, but long‐term outcomes remain dismal [3, 4]. Similarly, patients with refractory DLBCL have a median OS of approximately 6 months [5]. Outcomes in patients with MCL have improved with the development of Bruton's tyrosine kinase (BTK) inhibitors such as ibrutinib, but in patients with disease that has relapsed after receiving this therapy median OS ranges from 6 to 10 months [6]. In multiple myeloma (MM) the development of anti‐CD38 monoclonal antibodies has significantly improved the outcome for patients with disease that has relapsed after treatment with proteasome inhibitors and immunomodulatory agents. However, for patients who have progressed through all three classes of treatment outcomes are poor, with a median OS of approximately 9 [7]. These findings served as the rationale for clinical trials evaluating CAR T therapy in these malignancies (Table 1).

**TABLE 1:** Use of bridging therapy in CAR T clinical trials

Disease	Study/product	Total number of patients enrolled/infused	Numberof patients receiving bridging (%)	Bridging therapy (number of patients receiving)	Time to manufacture (median, range)	Comments
B cell ALL	ELIANA (Tisagenlecleucel – Kymriah) (NCT02435849)	92/75	65 (87)	Not specified	23 (range not available) days	Median time from enrollment to infusion 45 days (range 30–105). Patients received a median of three prior lines of therapy
B cell ALL	ZUMA‐3 (Brexucabtagene autoleucel – Tecartus) (NCT02614066)	71/55	51 (93)	Combinations of dexamethasone, vincristine, doxorubicin, mercaptopurine, hydroxyurea, methotrexate, fludarabine, cytarabine, idarubicin, cyclophosphamide, +/‐tyrosine kinase inhibitor	13 (11–14) days for U.S. patients	
MCL	ZUMA‐2 (Brexucabtagene autoleucel – Tecartus) (NCT02601313)	74/68	25 (37)	Steroids (14), ibrutinib (14), acalabrutinib (5), or combination (6)	16 (11–28) days	Seventeen patients with imaging assessments before and after – majority had increase in tumor burden; three patients died of progressive disease before treatment
Indolent lymphoma	ZUMA‐5 (Axicabtagene ciloleucel – Yescarta) (NCT03105336)	Unknown/146				Only primary efficacy analysis has been reported
High ‐grade B cell lymphoma	ZUMA‐1 (Axicabtagene ciloleucel – Yescarta) (NCT02348216)	111/101	0 (0)	Not allowed	17 (range not available) days	Ten patients did not receive treatment, one due to disease progression.
High ‐grade B cell lymphoma	JULIET (Tisagenlecleucel – Kymriah) (NCT02445248)	165/111	Not reported (92)	Combinations of rituximab, gemcitabine, etoposide, steroids, cisplatin, cytarabine, ibrutinib, lenalidomide	23 (range not available) days	Median time from enrollment to infusion 54 days (range 30–92 days), cryopreserved product used
High ‐grade B cell lymphoma	TRANSCEND (Lisocabtagene maraleucel) (NCT02631044)	344/269	159 (59)	Combinations of rituximab, gemcitabine, oxaliplatin, steroids, bendamustine, lenalidomide, brentuximab vedotin, ibrutinib	24 (17–51) days	Median time from leukapheresis to infusion 37 days (range 27–224). Patients with higher tumor burden and those who received bridging had higher rates of CRS and neurotoxicity.
Multiple myeloma	Idecabtagene vicleucel (NCT03361748)	140/128	112 (88)	Dexamethasone, cyclophosphamide, daratumumab, carfilzomib, bortezomib, pomalidomide	15 (1–33)	5/112 (4%) patients who received bridging therapy responded; no complete responses were reported.

Tisagenlecleucel (Tisa‐cel, Kymriah^©^) was the first CD19 CAR T therapy to be approved for use in children and young adults with relapsed/refractory ALL by the U.S. Food and Drug Administration (FDA) in 2017. A phase 2 clinical trial showed an overall remission rate of 81% at 3‐month and overall survival 76% at 1‐year after tisa‐cel administration for relapsed/refractory B cell ALL [8]. Later that year axicabtagene ciloleucel (axi‐cel; Yescarta^©^) was approved for patients with relapsed/refractory DLBCL after a trial showed an overall response rate of 82% and median OS at 12 months of 59% [9]. Subsequently, tisa‐cel and isocabtagene maraleucel (liso‐cel; Breyanzi^©^) were approved relapsed/refractory DLBCL. Brexucabtagene autoleucel (brexu‐cel, Tecartus^©^) were approved for use in relapsed/refractory MCL in 2020, and axi‐cel and idecabtagene vicleucel (ide‐cel, Abecma^©^) were approved for relapsed/refractory follicular lymphoma and relapsed/refractory MM, respectively in 2021 [10, 11, 12, 13].

Despite these advances, however, eligible patients face several challenges to receiving CAR T treatment, not least of which is progressive malignancy. In this paper, we review the barriers that arise when treating patients with CAR T therapy, evaluate the role of bridging therapy, and discuss recommendations for the use of bridging therapy in the future.

## 2 BARRIERS TO CAR T AND IMPORTANCE OF BRIDGING THERAPY

While the approval of various CAR T products has greatly expanded the treatment options available for patients with these aggressive malignancies, barriers remain that prevent patients from receiving this potentially curative therapy. These barriers range from social to economic to medical and can lead to significant delays or entirely prevent patients from being treated.

Social issues that contribute to delays in being treated are often financial or insurance‐related. The cost of CAR T therapy is significant, with the product alone costing approximately $400,000, which does not include the cost of inpatient hospitalization and treatment of complications that may arise [14]. While Centers for Medicare and Medicaid Services (CMS) recently increased coverage for CAR T treatment reimbursement remains well below the cost of therapy, particularly when factoring in additional costs [15]. Similar coverage issues are faced by patients with private insurance and can lead to delays in receiving necessary therapy for some patients.

Currently, the majority of CAR T therapies are administered in the inpatient setting, requiring hospitalization that may be prolonged depending on the development of toxicity. Recent studies have demonstrated the safety and feasibility of administering CAR T in the outpatient setting, but this creates new logistical issues [16, 17]. These patients were required to have a caregiver with them throughout therapy and were required to stay nearby the treating facility for the first month after CAR T administration. Problems can arise with both inpatient and outpatient treatment that can lead to delays or problems with receiving the therapy.

Additional logistical barriers can arise during apheresis and manufacturing of CAR T. Collection of patient's peripheral blood mononuclear cells occurs via apheresis and successful collection is dependent on the patient having adequate circulating lymphocytes. Difficulties can arise in patients who have recently been treated with chemotherapy that has depleted their lymphocyte count or in patients who have persistently low lymphocyte counts due to prior lines of therapy. In leukapheresis experience from clinical trials, the manufacturing process was unsuccessful in anywhere from 4% to 7% of patients and in one study that specifically evaluated leukapheresis for CAR T four of 41 patients required a second leukapheresis for adequate manufacturing [9, 18, 19, 20]. The manufacturing process requires activation of these T cells in cell culture, transduction or electroporation to introduce the CAR gene and ex vivo expansion of CAR T cells. The time from the apheresis to CAR T cell delivery in clinical trials ranged from 16 to 24 days. One multicenter retrospective review reported the median time from apheresis to CAR T infusion at 28 days for axi‐cel and 44 days for tisa‐cel; in the JULIET trial time from apheresis to infusion ranged from 30 to 92 days [10, 21].

Finally, problems with receiving CAR T therapy can be related to rapidly progressive disease. In clinical trials between 1 to 4% of patients died from disease progression before receiving CAR T therapy [9, 11]. In addition, in real‐world experience with patients with non‐Hodgkin lymphoma who were treated with axi‐cel, bulky disease, defined as tumor > 10 cm, were at higher risk for developing toxicity during CAR T treatment. Patients with lactate dehydrogenase elevations before treatment were also noted to have shorter progression‐free survival [22]. Hence, bridging therapy serves two purposes: to control disease while waiting for CAR T infusion and to reduce the risk of CAR T‐associated toxicities by debulking the disease.

## 3 PREVIOUS EXPERIENCE WITH BRIDGING THERAPY

Several clinical trials that served as the basis for approval for various CAR T products allowed the use of bridging therapy but bridging therapy in these trials was diverse and reported with a varying degree of detail.

The ELIANA trial (NCT02435849), which led to the approval of tisa‐cel for patients with B‐cell ALL allowed the use of bridging therapy. Of the total 92 patients who were enrolled in the trial 65 received bridging therapy although details of regimens used for bridging were not specified median time to manufacture in this trial was reported at 23 days. The product was cryopreserved, and the median time from enrollment to infusion was 45 days (range 30–105 days) [8].

Bridging therapy was not allowed in ZUMA‐1 (NCT02348216) trial evaluating axi‐cel in DLBCL. Of note, the median time to manufacture in this trial was 17 days and of 10 the patients who did not receive treatment one of them died due to disease progression [9]. The JULIET (NCT02445248) trial evaluating tisa‐cel in DLBCL allowed bridging therapy, which was given in 92% of enrolled patients (number of patients receiving bridging was not reported) [10]. The choice of bridging was diverse, including combinations of rituximab, gemcitabine, etoposide, steroids, cisplatin, cytarabine, ibrutinib, and lenalidomide. The median time to manufacture in this trial was 23 (range not available) days. Though the exact numbers were not included, it was reported that the majority of the 50 patients who enrolled in the study but discontinued participation before receiving CAR T did so due to progressive disease or death. A subgroup analysis of seven patients from the JULIET trial who were excluded from the original efficacy data who had a complete response to bridging therapy and subsequently received CAR T infusion has also been published [20]. In this analysis, the duration of bridging therapy ranged from 2 days to 129 days, and a variety of regimens were used. Of these patients, five remained progression‐free for more than 12 months, and this group of patients was found to have low rates of cytokine release syndrome (CRS) and neurotoxicity.

The TRANSCEND (NCT02631044) study evaluated the use of liso‐cel in patients with DLBCL and bridging therapy was allowed. Of the 344 patients enrolled, 159 (59%) received bridging therapy which included combinations of rituximab, gemcitabine, oxaliplatin, steroids, bendamustine, lenalidomide, brentuximab vedotin, and ibrutinib. Fourty‐eight of 344 patients who underwent leukapheresis had complications of disease or died before receiving CAR T infusion. In most patients, bridging therapy did not result in lower disease burden and a higher tumor burden and receipt of bridging therapy were associated with higher rates of CRS and neurotoxicity. The median time to manufacture was the highest in this trial, reported at 24 (17–51) days [19].

The ZUMA‐2 trial (NCT02601313) evaluating brexu‐cel in MCL included 74 patients of which 25 (35%) received bridging therapy. Bridging for these patients included steroids, ibrutinib, acalabrutinib, or a combination. Of the 25 patients who received bridging, 17 had imaging assessments before and after, and the majority of those patients were found to have an increase in tumor burden. Three patients died of progressive disease before receiving CAR T treatment. The median time to manufacture was reported as 16 (11–28) days [11].

The KarMMa trial (NCT03361748) evaluated the use of ide‐cel in MM enrolled 178 patients, of whom 112 (88%) received bridging therapy. Bridging therapy in these patients included dexamethasone, cyclophosphamide, daratumumab, carfilzomib, bortezomib, and pomalidomide. Disease reassessments performed after bridging and before CAR T treatment showed that five patients had responded to bridging therapy.

Some data are also available regarding the use of bridging therapy with CAR T in commercial use. A study published by the U.S. Lymphoma CAR T Consortium evaluated the use of axi‐cel for DLBCL in 298 patients across 17 centers [22]. Unlike the ZUMA‐1 trial, bridging therapy was used in 158 (53%) of patients and consistent of combinations of steroids, chemotherapy, radiation, or targeted therapies such as lenalidomide or ibrutinib. A multivariate analysis from this study demonstrated worse overall survival at 12 months in patients who received bridging therapy (56% vs. 81% in patients who did not receive bridging therapy, *p* < 0.001). Another study evaluated the influence of bridging therapy on outcomes in 148 patients with DLBCL who were being treated with axi‐cel. Eighty‐one patients (55%) received bridging, whether in the form of systemic therapy, radiation therapy, or combined‐modality therapy [23]. The patients receiving bridging were more likely to have an elevated international prognostic index score, bulky disease, and an elevated lactate dehydrogenase (LDH). Overall survival at 1 year was significantly different in patients receiving bridging therapy, reported at 48% (vs. 65% in patients who did not receive bridging, *p* = 0.05). Of note, in this study radiation therapy (RT) alone was shown to be a safe and effective bridging strategy in a small cohort (*n* = 11) of patients. These patients who were bridged with RT had similar rates of toxicity as patients who received systemic bridging or no bridging and had an improved progression‐free survival compared to patients who were bridged with systemic therapy. In a small study, 12 patients were treated with radiation therapy as bridging before receiving axi‐cel and radiation was shown to be safe and response rates and complication rates after axi‐cel were similar to those in the original study [24].

Two retrospective studies have also evaluated the use of bridging therapy. The first evaluated 46 patients who were receiving commercial CAR T products and included 30 (65%) who received high‐intensity bridging therapy, defined as chemotherapy with or without immunotherapy, and 16 (35%) who received low intensity or no bridging therapy [25]. In this study, the intensity of bridging treatment was closely related to tumor burden at enrollment and while there was no difference in efficacy of CAR T infusion between the high‐intensity and low‐intensity groups, similar to other studies the high‐intensity group did have a higher frequency of CRS and neurotoxicity. Another single‐institution retrospective study evaluated 64 patients with non‐Hodgkin lymphoma, 49 of whom received commercial CAR T [26]. Thirty‐four (69%) of these 49 patients received bridging therapy to reduce tumor burden or palliate symptoms. Bridging therapies included combination chemoimmunotherapy, radiation alone, systemic therapy with radiation therapy, targeted treatments, or combination treatment. Of the patients who received chemoimmunotherapy, three of 12 had progressive disease at the time of CAR T infusion and of five patients who received radiation alone, one had progressive disease at the time of CAR T infusion.

## 4 CLINICAL APPLICATION OF BRIDGING THERAPY

CAR T therapy is safe and effective in a variety of aggressive relapsed and refractory malignancies. Studies have shown that patients with a lower burden of disease at time of treatment tend to have lower rates of toxicity and significantly better survival, with one study showing median overall survival of 20.1 months in patients with low disease burden at time of treatment (vs. 12.9 months in the total cohort) [27, 28].

In clinical trials, data regarding the effects of bridging on disease burden are mixed, and several studies have demonstrated that the use of bridging therapy is associated with higher rates of toxicity and poorer overall survival. This is likely reflective of the fact that bridging therapy is often used in patients with more aggressive disease, a higher burden of disease at baseline, or disease that is refractory to chemotherapy. We also do not have information about incorporating novel agents (blinatumomab, polatuzumab vendotin, loncastuximab tesirine), which may have better efficacy in debulking disease compared to traditional chemoimmunotherapy or radiation. Additional studies and longer follow‐up are needed to fully characterize the optimal regimens, duration, and timing for bridging therapy.

Bridging therapy should be considered in patients with an aggressive and high burden disease and should be when delays in treatment are expected, whether due to financial, social, or manufacturing issues. In these patients, it is important to confirm that the leukapheresed cells have been received by the manufacturing facility and are adequate for manufacturing prior to starting bridging therapy in case additional apheresis is required. However, the use and timing of bridging therapy needs to be balanced with the urgency of administering definitive CAR T treatment. Bridging therapy has been used safely with all the commercially available products regardless of whether it was used in clinical trials, so can be considered independent of which product is chosen.

The choice of bridging therapy should be carefully considered (Table 2). Prior regimens and side effects from prior treatment should be evaluated, and therapies should be chosen that avoid worsening any short‐ or long‐term side effects. Consideration should be given to avoiding regimens that may cause significant myelosuppression. Avoidance of regimens that may lead to significant lymphopenia is critical, and nucleoside analogs should be avoided if possible given their importance as lymphodepleting therapy prior to CAR T infusion and risk of prolonged immunosuppression and infectious complications. Intensive chemotherapy is not necessarily superior when used as bridging therapy, and targeted agents can be safely used in selected cases. Additionally, in patients who are candidates, radiation therapy should be considered as it has been used safely and avoids many of the side effects of systemic bridging therapy.

**TABLE 2:** Considerations for bridging therapy

Disease	When to consider bridging therapy	Choices for bridging therapy
NHL	Bulky disease (≥10 cm), > 1 extranodal site involved, stage 3–4 disease, bone marrow involvement, elevated pretreatment LDH, CRP	Rituximab ± chemotherapy (gemcitabine, etoposide, cisplatin, cytarabine, bendamustine, oxaliplatin) Polatuzumab (± bendamustine, rituximab) Single agent: lenalidomide, BTK inhibitor, Tafasitamab^a^ ± Steroids ± XRT
B cell ALL	Bone marrow blasts > 5%, extramedullary disease, CNS disease^b^	Chemotherapy (single agent or combination): vincristine, doxorubicin/idarubicin, mercaptopurine, methotrexate, fludarabine, cytarabine, cyclophosphamide Single agent: TKI, hydroxyurea, inotuzumab ozogamicin, blinatumomab^c^ ± Steroids
MM	Bone marrow involvement > 50%, extramedullary disease, R‐ISS stage III disease	Single agent or combination: cyclophosphamide, pomalidomide, bortezomib, carfilzomib, melflufen, belantamab mafodotin^d^ : anti‐CD38 mAb, + steroids ± XRT

Abbreviations: ALL, acute lymphoblastic leukemia; BTK, Bruton's tyrosine kinase; LDH, lactate dehydrogenase; MM, multiple myeloma; NHL, non‐Hodgkin lymphoma; TKI, tyrosine kinase inhibitor; XRT, radiation therapy; CSF, cerebrospinal fluid.

^a^ Also targets CD19, safety and effects when used as bridging to CAR T are unknown.

^b^ Consider intrathecal therapy to clear CSF disease.

^c^ Bispecific T cell engager targeting CD19 may be used as bridging therapy if CD19 expression remains adequate.

^d^ Targets B cell manuration antigen (BCMA), safety, and effects when used as bridge to anti‐BCMA CAR T are unknown.

These considerations should be balanced against the possible pitfalls of using bridging therapy. In addition to using regimens that avoid worsening previously existing side effects from therapy, side effects from bridging therapy may delay infusion, particularly if they cause significant myelosuppression, infections, or significant organ toxicities. Data regarding the use of regimens that target the same tumor antigen as the CAR T is mixed and requires further evaluation. In the ZUMA‐3, which evaluated brexu‐cel in patients with relapsed/refractory B cell ALL, patients who had received prior blinatumomab were included if their leukemic blasts had >90% CD19 expression [29]. In a subgroup analysis, rates of relapse free survival (RFS) and OS were similar among patients who had received prior blinatumomab. Conversely, in a phase I study of a CD22‐targeted CAR T cell product, patients who had previously received anti‐CD22 targeted therapy (including 14 patients with prior inotuzumab ozogamicin exposure) were found to have decreased minimal residual disease (MRD)‐negative CR rates and shorter remission durability than those who did not [30]. Finally, care should be taken to avoid bridging therapy with a prolonged half‐life, which may negatively impact CAR T activity.

Ultimately, the decision to proceed with bridging therapy should be individualized for each patient and should involve the systematic evaluation of the following factors: tumor burden, number and type of prior lines of treatment, and expected time from apheresis to infusion of CAR T. In the future, the development and rapid availability of off the shelf CAR T products may eliminate the need for bridging therapy.

## CONFLICTS OF INTEREST

STB, SMS and BNS report no COI. BRD reports institutional funding from Takeda, Janssen, Angiocrine, Pfizer, Poseida, MEI, Sorrento. Consultancy with Jazz, Celgene, Gamida Cell. OOO reports advisory board consultancy with Pfizer, Novartis, Janssen, Kite, Gilead, Spectrum, Bayer and Curio science. Research funding with Kite.

## References

[1] Till BG , Jensen MC , Wang J , Chen EY , Wood BL , Greisman HA , et al. Adoptive immunotherapy for indolent non‐hodgkin lymphoma and mantle cell lymphoma using genetically modified autologous CD20‐specific T cells. Blood. 2008;112(6):2261‐71. 10.1182/blood-2007-12-128843 18509084 PMC2532803

[2] Kalos M , Levine BL , Porter DL , Katz S , Grupp SA , Bagg A , et al. T cells with chimeric antigen receptors have potent antitumor effects and can establish memory in patients with advanced leukemia. Sci Transl Med. 2011;3(95):95ra73‐95ra73. 10.1126/scitranslmed.3002842 PMC3393096 21832238

[3] Gökbuget N , Stanze D , Beck J , Diedrich H , Horst H‐A , Hüttmann A , et al. Outcome of relapsed adult lymphoblastic leukemia depends on response to salvage chemotherapy, prognostic factors, and performance of stem cell transplantation. Blood. 2012;120(10):2032‐41. 10.1182/blood-2011-12-399287 22493293

[4] Kantarjian H , Stein A , Gökbuget N , Fielding AK , Schuh AC , Ribera J‐M , Wei A , et al. Blinatumomab versus chemotherapy for advanced acute lymphoblastic leukemia. N Engl J Med. 2017;376(9):836‐47. 10.1056/NEJMoa1609783 28249141 PMC5881572

[5] Crump M , Neelapu SS , Farooq U , Van Den Neste E , Kuruvilla J , Westin J , et al. Outcomes in refractory diffuse large B‐cell lymphoma: Results from the international SCHOLAR‐1 study. Blood. 2017;130(16):1800‐8. 10.1182/blood-2017-03-769620 28774879 PMC5649550

[6] Jain P , Kanagal‐Shamanna R , Zhang S , Ahmed M , Ghorab A , Zhang L , et al. Long‐term outcomes and mutation profiling of patients with mantle cell lymphoma (MCL) who discontinued ibrutinib. Br J Haematol. 2018;183(4):578‐87. 10.1111/bjh.15567 30175400

[7] Gandhi UH , Cornell RF , Lakshman A , Gahvari ZJ , Mcgehee E , Jagosky MH , et al. Outcomes of patients with multiple myeloma refractory to CD38‐targeted monoclonal antibody therapy. Leukemia. 2019;33(9):2266. 10.1038/S41375-019-0435-7 30858549 PMC6820050

[8] Maude SL , Laetsch TW , Buechner J , Rives S , Boyer M , Bittencourt H , et al. Tisagenlecleucel in children and young adults with B‐cell lymphoblastic leukemia. N Engl J Med. 2018;378(5):439‐48. 10.1056/NEJMoa1709866 29385370 PMC5996391

[9] Neelapu SS , Locke FL , Bartlett NL , Lekakis LJ , Miklos DB , Jacobson CA , et al. Axicabtagene ciloleucel CAR T‐cell therapy in refractory large B‐Cell lymphoma. N Engl J Med. 2017;377(26):2531‐44. 10.1056/NEJMoa1707447 29226797 PMC5882485

[10] Schuster SJ , Bishop MR , Tam CS , Waller EK , Borchmann P , Mcguirk JP , et al. Tisagenlecleucel in adult relapsed or refractory diffuse large B‐cell lymphoma. N Engl J Med. 2019;380(1):45‐56. 10.1056/NEJMoa1804980 30501490

[11] Wang M , Munoz J , Goy A , Locke FL , Jacobson CA , Hill BT , et al. KTE‐X19 CAR t‐cell therapy in relapsed or refractory mantle‐cell lymphoma. N Engl J Med. 2020;382(14):1331‐42. 10.1056/NEJMoa1914347 32242358 PMC7731441

[12] Jacobson C , Chavez JC , Sehgal AR , William BM , Munoz J , Salles G , et al. Primary analysis of Zuma‐5: a phase 2 study of axicabtagene ciloleucel (Axi‐Cel) in patients with relapsed/refractory (R/R) indolent non‐Hodgkin lymphoma (iNHL). Blood. 2020;136(Supplement 1):40‐1. 10.1182/blood-2020-136834

[13] Munshi NC , Anderson LD , Shah N , Madduri D , Berdeja J , Lonial S , et al. Idecabtagene vicleucel in relapsed and refractory multiple myeloma. N Engl J Med. 2021;384(8):705‐16. 10.1056/nejmoa2024850 33626253

[14] Bach PB . National coverage analysis of CAR‐T therapies—policy, evidence, and payment. N Engl J Med. 2018;379(15):1396‐8. 10.1056/NEJMp1807382 30110578

[15] Centers for Medicare & Medicaid Services . Fiscal Year (FY) 2020 Medicare Hospital Inpatient Prospective Payment System (IPPS) and Long Term Acute Care Hospital (LTCH) Prospective Payment System (CMS‐1716‐F) | CMS. https://www.cms.gov/newsroom/fact‐sheets/fiscal‐year‐fy‐2020‐medicare‐hospital‐inpatient‐prospective‐payment‐system‐ipps‐and‐long‐term‐acute‐0. Accessed April 8, 2021.

[16] Bachier CR , Godwin JE , Andreadis C , Palomba ML , Abramson JS , Sehga AR , et al. Outpatient treatment with lisocabtagene maraleucel (liso‐cel) across a variety of clinical sites from three ongoing clinical studies in relapsed/refractory (R/R) large B‐cell lymphoma (LBCL). J Clin Oncol. 2020;38(15_suppl):8037‐8037. 10.1200/jco.2020.38.15_suppl.8037

[17] Alexander M , Culos K , Roddy J , Shaw JR , Bachmeier C , Shigle TL , et al. Chimeric antigen receptor T cell therapy: a comprehensive review of clinical efficacy, toxicity, and best practices for outpatient administration. Transplant Cell Ther. 2021;27(7):558‐70. 10.1016/J.JTCT.2021.01.014 33910041

[18] Korell F , Laier S , Sauer S , Veelken K , Hennemann H , Schubert M‐L , et al. Current challenges in providing good leukapheresis products for manufacturing of cAR‐T cells for patients with relapsed/refractory NHL or ALL. Cells. 2020;9(5):1225. 10.3390/cells9051225 PMC7290830 32429189

[19] Abramson JS , Palomba ML , Gordon LI , Lunning MA , Wang M , Arnason J , et al. Lisocabtagene maraleucel for patients with relapsed or refractory large B‐cell lymphomas (TRANSCEND NHL 001): a multicentre seamless design study. Lancet. 2020;396(10254):839‐52. 10.1016/S0140-6736(20)31366-0 32888407

[20] Bishop MR , Maziarz RT , Waller EK , Jäger U , Westin JR , Mcguirk JP , et al. Tisagenlecleucel in relapsed/refractory diffuse large B‐cell lymphoma patients without measurable disease at infusion. Blood Adv. 2019;3(14):2230‐6. 10.1182/bloodadvances.2019000151 31332046 PMC6650727

[21] Riedell PA , Walling C , Nastoupil LJ , Pennisi M , Maziarz RT , Mcguirk JP , et al. A multicenter retrospective analysis of clinical outcomes, toxicities, and patterns of use in institutions utilizing commercial axicabtagene ciloleucel and tisagenlecleucel for relapsed/refractory aggressive B‐Cell lymphomas. Blood. 2019;134(Supplement_1):1599. 10.1182/blood-2019-127490

[22] Nastoupil LJ , Jain MD , Feng L , Spiegel JY , Ghobadi A , Lin Yi , et al. Standard‐of‐care axicabtagene ciloleucel for relapsed or refractory large B‐cell lymphoma: Results from the US lymphoma CAR T consortium. J Clin Oncol. 2020;38(27):3119‐28. 10.1200/JCO.19.02104 32401634 PMC7499611

[23] Pinnix CC , Gunther JR , Dabaja BS , Strati P , Fang P , Hawkins MC , et al. Bridging therapy prior to axicabtagene ciloleucel for relapsed/refractory large B‐cell lymphoma. Blood Adv. 2020;4(13):2871‐83. 10.1182/bloodadvances.2020001837 32589728 PMC7362355

[24] Sim AJ , Jain MD , Figura NB , Chavez JC , Shah BD , Khimani F , et al. Radiation therapy as a bridging strategy for CAR T cell therapy with axicabtagene ciloleucel in diffuse large B‐cell lymphoma. Int J Radiat Oncol Biol Phys. 2019;105(5):1012‐1021. 10.1016/j.ijrobp.2019.05.065 31175906 PMC6872916

[25] Paillassa J , Vercellino L , Di Blasi R , Bernard S , Moatti H , Bommier C , et al. Impact of bridging chemotherapy on clinical outcomes of CD19 CAR T therapy in relapse/refractory diffuse large B‐ cell lymphoma in real world experience. Blood. 2019;134(Supplement_1):2886. 10.1182/blood-2019-129421

[26] Dwivedy Nasta S , Hughes ME , Namoglu EC , Landsburg DJ , Chong EA , Barta SK , et al. A characterization of bridging therapies leading up to commercial CAR T‐cell therapy. Blood. 2019;134(Supplement_1):4108. 10.1182/blood-2019-131399

[27] Hay KA , Hanafi L‐A , Li D , Gust J , Liles WC , Wurfel MM , et al. Kinetics and biomarkers of severe cytokine release syndrome after CD19 chimeric antigen receptor–modified T‐cell therapy. Blood. 2017;130(21):2295‐306. 10.1182/blood-2017-06-793141 28924019 PMC5701525

[28] Park JH , Rivière I , Gonen M , Wang X , Sénéchal B , Curran KJ , et al. Long‐term follow‐up of CD19 CAR therapy in acute lymphoblastic leukemia. N Engl J Med. 2018;378(5):449‐59. 10.1056/NEJMoa1709919 29385376 PMC6637939

[29] Shah BD , Ghobadi A , Oluwole OO , Logan AC , Boissel N , Cassaday RD , et al. KTE‐X19 for relapsed or refractory adult B‐cell acute lymphoblastic leukaemia: phase 2 results of the single‐arm, open‐label, multicentre ZUMA‐3 study. Lancet. 2021;398(10299):491‐502. 10.1016/S0140-6736(21)01222-8 34097852 PMC11613962

[30] Shah NN , Highfill SL , Shalabi H , Yates B , Jin J , Wolters PL , et al. CD4/CD8 T‐Cell selection affects chimeric antigen receptor (CAR) T‐cell potency and toxicity: updated results from a phase I anti‐CD22 CAR T‐cell trial. J Clin Oncol. 2020;38(17):1938‐50. 10.1200/JCO.19.03279 32286905 PMC7280047

